# Editorial: pacing after transcatheter tricuspid valve replacement: where are we going, where do we stand?

**DOI:** 10.1093/ehjcr/ytag049

**Published:** 2026-01-25

**Authors:** Fabian Barbieri, Vasileios Exarchos, Markus Reinthaler, Johannes Lucas

**Affiliations:** Deutsches Herzzentrum der Charité, Department of Cardiology, Angiology and Intensive Care Medicine, Campus Benjamin Franklin, Hindenburgdamm 30, Berlin 12203, Germany; Charité – Universitätsmedizin Berlin, corporate Member of Freie Universität Berlin and Humboldt-Universität zu Berlin, Department of Cardiology, Hindenburgdamm 30, Berlin 12203, Germany; Institute of Active Polymers and Berlin-Brandenburg Center for Regenerative Therapies, Helmholtz-Zentrum Hereon, Kantstraße 55, Teltow 14513, Germany; Deutsches Herzzentrum der Charité, Department of Cardiology, Angiology and Intensive Care Medicine, Campus Benjamin Franklin, Hindenburgdamm 30, Berlin 12203, Germany; Charité – Universitätsmedizin Berlin, corporate Member of Freie Universität Berlin and Humboldt-Universität zu Berlin, Department of Cardiology, Hindenburgdamm 30, Berlin 12203, Germany; Deutsches Herzzentrum der Charité, Department of Cardiology, Angiology and Intensive Care Medicine, Campus Benjamin Franklin, Hindenburgdamm 30, Berlin 12203, Germany; Charité – Universitätsmedizin Berlin, corporate Member of Freie Universität Berlin and Humboldt-Universität zu Berlin, Department of Cardiology, Hindenburgdamm 30, Berlin 12203, Germany; Institute of Active Polymers and Berlin-Brandenburg Center for Regenerative Therapies, Helmholtz-Zentrum Hereon, Kantstraße 55, Teltow 14513, Germany; Deutsches Herzzentrum der Charité, Department of Cardiology, Angiology and Intensive Care Medicine, Campus Benjamin Franklin, Hindenburgdamm 30, Berlin 12203, Germany; Charité – Universitätsmedizin Berlin, corporate Member of Freie Universität Berlin and Humboldt-Universität zu Berlin, Department of Cardiology, Hindenburgdamm 30, Berlin 12203, Germany

**This editorial refers to ‘Transjugular leadless pacemaker implantation after transcatheter tricuspid valve replacement with a biological prosthetic valve: a case report’ by M. Fortuna *et al*., https://doi.org/10.1093/eurheartj/ytaf609**.

The field of transcatheter tricuspid valve interventions (TTVI) is evolving quickly with many devices and concepts being currently evaluated in first in human trials and early feasibility studies.^1^ Up to date, only few of them have been cleared by FDA or obtained Ce mark. Besides the well-established transcatheter edge-to-edge repair, a surge of transcatheter tricuspid valve replacement (TTVR) is observed since the presentation of the TRISCEND II data evaluating the EVOQUE^®^ valve (Edwards Lifesciences, Irvine, CA, USA). The device showed excellent therapeutical results regarding nihilation of tricuspid regurgitation and short-term symptomatic improvement with the longest available data reaching the 12-month mark also leading to regulatory approval.^2^ Nonetheless, a considerable number of patients (10–25%) require permanent pacemaker (PPM) therapy due to the occurrence of higher degree atrioventricular block (HAVB).^2–4^

## Predictors of higher degree atrioventricular block and pathophysiology

Most important predictors of HAVB are preexisting left bundle branch or left fascicular blocks as well as device oversizing while around 20% of patients develop a new-onset right bundle branch block.^5^ Besides direct trauma to the atrioventricular node, which usually occurs during the very first postprocedural days, the occurrence of HAVB may also be delayed and seen up to 30 days post implantation. This may reflect the increasing trauma by the rigid prosthesis due to successive ventricular offloading with consecutive structural remodelling leading to a gradual increase of tension opposing to the septal area.

## Treatment options

Overall, there are many concepts available to adequately provide ventricular pacing in patients with HAVB (*Table 1*), but the presence of the transcutaneously implanted tricuspid valve marks an additional challenge for physicians by representing itself as an obstacle to either surpass or work around. Conventional lead-based strategies include epicardial lead placement, either via the coronary sinus or by surgical means, and transvalvular lead placement. Unfortunately, each strategy opposes some disadvantages, especially in the frail and aged patient cohort of patients with significant tricuspid regurgitation, resulting in an ongoing search for alternatives. In the presented case, the authors highlighted the difficulties of implanting a leadless cardiac pacemaker (LCPM) system in a patient with previous TTVR by transfemoral access due to limited working height.^6^ By changing to transjugular access, an easier angulation between the venae cavae and the tricuspid annulus plane was observed and facilitated passage, while also improving manoeuvrability to allow successful placement of a LCPM in adequate position.

**Table 1:** Concepts for ventricular pacing and their potential role in patients with transcatheter tricuspid valve replacement

Pacing strategy	Potential advantages	Potential disadvantages
Single left ventricular lead via coronary sinus	No interaction between prosthesis and lead More physiological form of pacing	Level of asynchronous pacing remains Higher risk of lead dislodgement Higher stimulation threshold leading to faster battery depletion and more frequent box exchanges Phrenic stimulation
Epicardial leads (surgical)	Extravenous hardware No interference between leads and prosthesis Lower risk for endocarditis Cardiac resynchronization therapy possible	Invasive
Transvenous right ventricular lead through prosthesis	Simple Cost-effective	Lead-prosthesis interference Recurrence of tricuspid regurgitation by functional impairment of prosthesis due to transprosthetical lead
Conventional leadless pacemaker (transfemoral access)	No interference between device and prosthesis after successful implantation Reduced hardware Very low risk for infection	Inferior vena cava to right atrium angulation with impaired manoeuvrability Tilting/partial dislocation of the prosthesis while passaging the valve with delivery catheter Right ventricular pacing only/asynchronous pacing with conventional devices Costs
Conventional leadless pacemaker (transjugular access)	No interference between device and prosthesis after successful implantation Reduced hardware Superior vena cava to right atrium angulation Improved manoeuvrability compared to transfemoral access Very low risk for infection	Tilting/partial dislocation of the prosthesis while passaging the valve with delivery catheter Right ventricular pacing only/asynchronous pacing with conventional devices Costs
Physiologic pacing by leads (His bundle or left bundle branch area pacing)	Most physiologic form of ventricular pacing with all potential advantages Cost-effective	Target zone often blocked by valve prosthesis Lead-prosthesis interference Recurrence of tricuspid regurgitation by functional impairment of prosthesis in case of transprosthetical lead

The general feasibility and safety of transjugular LCPM implantation was already described by Saleem-Talib *et al*. with the MICRA® system (Medtronic, Mineapolis, MN, USA) in patients without any form of previous TTVI. Here, the improved steering of the catheter led to a non-apical placement of the LCPM in about 80% yielding a narrower QRS width, reducing the amount of asynchrony in right ventricular pacing with all its potential downsides.^7^ Compared to this analysis, Fortuna *et al*. used the AVEIR^®^ system (Abbott Laboratories, Abbott Park, IL, USA), which also offers a dedicated sheath for transjugular access.^6^ Alternatives to the transjugular access are the left subclavian vein although only a single case of successful LCPM implantation is described in the literature.^8^ Unfortunately, conventional leadless PPM devices only deliver right ventricular pacing, which is known to be more asynchronous than intrinsic conduction with all potential downsides.^9^ Newer concepts, like Wireless Stimulation Endocardially for Cardiac Resynchronization (WiSE-CRT^®^, EBR Systems Santa Clara, CA, USA), could enable more physiological ways of pacing especially in patients with known left ventricular dysfunction or ones developing pacing-induced cardiomyopathy (PICM) by enabling a completely leadless system for cardiac resynchronization therapy.^10,11^ Recently published case reports also describe its potential value to provide left bundle branch area pacing.^12,13^

## Future perspective

Beyond above mentioned concepts, it remains to be seen whether new strategies for pacing will evolve because of a more widespread application of TTVR devices or newer TTVR devices will resolve the issue of HAVB occurrence with some of them already holding high promises.^14^ The optimism for TTVR seems nonetheless unbroken although a significant improvement in hard endpoints like mortality is yet to be observed.^2^ This could also be due to the negative effect of PPM therapy, which is now known to affect long-term outcomes in other forms of valvular therapy.^15^ Accordingly, it remains a top priority to fix this issue; otherwise, we may end up trading tricuspid regurgitation for PICM only to open another can of worms.

## Lead author biography


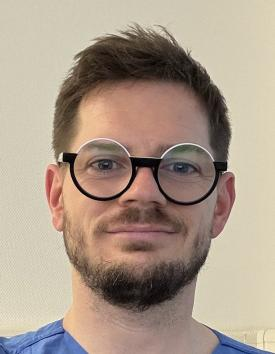


Fabian Barbieri currently works at Deutsches Herzzentrum der Charité. His main interests include structural heart diseases, cardiac implantable electronic device therapies and multimodality imaging.

**Funding:** None declared.
